# What Are the Indications at the Present Time for the Direct Tranfusion of Blood?

**Published:** 1911-03

**Authors:** E. G. Jones, D. F. Winn

**Affiliations:** Atlanta, Ga.; Atlanta, Ga.


### WHAT ARE THE INDICATIONS AT THE PRESENT TIME FOR THE DIRECT TRANSFUSION OF BLOOD?

E. G. Jones, M. D. and D. F. Winn, M. D., Atlanta, Ga.

The recent developments of Carrel, Crile, Guthrie Elsberg, and others in the suturing of blood vessels and in mechanical devices designed for a rapid, simple and safe technic for the direct transfusion of blood have put into our hands a measure of undoubted therapeutic value. The following conclusions in reference to the clinical uses of transfusion have been compiled from the recent literature upon the subject.

### HEMORRHAGE.

### Acute Hemorrhage.

In acute hemorrhage, where the source of the bleeding can be controlled, transfusion furnishes the ideal treatment, and has invariably been a life-saving measure when done by competent hands before irreparable damage has resulted from anemia in the central nervous system. While the performance is exceedingly difficult and tedious and should not be attempted without previous experience upon lower animals, no surgeon who is in position to carry out the technic should fail to be familiar with some method of transfusion—even if the relief of accidental hemorrhage were the only indication for its use.

### Hemorrhage from Bowels in Typhoid Fever.

Transfusion for hemorrhage in typhoid has been practiced a number of times successfully in cases in which the ordinary measures had failed, though in several cases the improvement has been only temporary, to be followed by hemorrhage at a subsequent time from which the patients died. One could scarcely expect blood transfusion to prevent hemorrhage from new points of ulceration in typhoid when the pathological process producing the bleeding is considered, but certainly this does not render the institution of transfusion at all illogical.

### Chronic Gastro-Intestinal Hemorrhage.

By transfusion in this condition, not only may the lost blood be replaced and the patient thus gain sufficient strength to warrant an operation directed towards hemostasis, but the effect of the donor's blood may even go so far as to prevent further bleeding and accomplish a cure when all other methods of treatment have been of no avail.

### Intractable Post-Operative Hemorrhage.

Here transfusion offers a resuscitation of the patient by a restoration of the lost blood so that radical steps can be taken to secure the bleeding point. If advisable the transfusion may be instituted concomitant with the search for the source of bleeding. Transfusion in some instances of post operative hemorrhage—notably in hemorrhage from kidney and bladder wall after nephrotomy—has seemed to exert an influence towards the spontaneous checking of the hemorrhage.

### Hemophilia.

Crile furnishes encouraging reports of hemophilia treated by the direct transfusion of blood. In every case treated there has been immediate temporary cessation of hemorrhage. In two of his cases hemorrhage soon recurred, to be followed by hemostasis.

Newell reports one case occurring in a new-born child in which immediate stoppage of hemorrhage occurred as soon as the effect of the donor's blood became evident in the recipient.

Goodman has recently reported a case of hemophilia in a child two and a half years old. Transfusion was considered as a last resort, calcium lactate, hypodermoclysis, pressure, astringents, etc. having proved futile. The hemorrhage stopped and the child began to improve immediately. The fact that the patient has since passed through a seige of measles and his scalp has been subjected to free incision and drainage for cellulitis appears to establish proof of a permanent cure in this case.

### Melena Neonatorum.

Until the introduction of transfusion of blood for this condition all the measures directed towards relieving it were entirely of no avail. Now, there is no doubt that by transfusion it is possible to save a large majority of these patients.

Transfusion in melena neonatorum seems to disprove both the infectious and the blood-vessel theory as to the etiology of this hemorrhage; one could hardly infer that the wonderful change from a moribund state to a state of perfect health, occurring within a few hours after transfusion, could be due to a regeneration of the capillaries or to a sudden convalescence from infection.

Successful results have also been reported in eight successive cases by Welch in which, instead of directly transfusing blood from donor to recipient, he has introduced sterile human blood serum subcutaneously in doses of 10 c. c. at a time and repeated after several hours if necessary. It is claimed that this procedure eliminates the drawback of obtaining a suitable donor and the difficulty of anastomosing extremely small vessels. We believe, however, that transfusion is more advantageous in that it stimulates the patient, replaces the lost blood blood and is equally effective at least as a curative measure.

PURPURA.

At the present time a definite conclusion cannot be formed in reference to the cure of purpura by transfusion, though from the work already done in this connection, it is probable that it will prove of value.

SECONDARY ANEMIA.

The success of Pool and McClure and others in transfusing for secondary anemia, even when the patient has been practically moribund, leads us to believe that this method of treatment has a well-established place in the therapy of this condition. There is no sort of doubt that many patients suffering with secondary anemia may be rendered safe surgical risks by the use of transfusion either before or during operation.

SHOCK.

By a series of interesting experiments in artificially produced shock, and in actual clinical cases, Crile has shown that transfusion acts almost as a specific in selected cases. By comparisons made under proper controls it has likewise been proven that transfusion is far superior to the intra-venous injection of normal saline for the reason that it is impossible for as great an amount of salt solution to be injected as the amount of blood which can be transfused without passing out of the recipient's vessels. Further, the transfusion of blood brings about a greater and more permanent rise in blood pressure than does the injection of artificial solutions.

Equally satisfactory results follow transfusion for combined hemorrhage and shock as has been experienced in shock from trauma, etc.

When there is any vitality at all remaining in the vasomotor centers they are revived by transfusion, so that the blood pressure —the extreme lowering of which constitutes the principal pathology of shock—is raised and maintained by two forces, viz., by the renewed activity of the vasomotor centers and by the mechanical effect on the heart of the increased volume of blood. As a prophylactic measure in preventing shock transfusion is distinctly indicated.

### Illuminating Gas Poisoning.

From experiments on dogs Crile found that about 60 per cent. seemed to be saved if the transfusion was accompanied by rythmic pressure on the thorax, artificial respiration and bleeding. Those cases treated by either simple manipulation, bleeding or saline transfusion all died.

### Pellagra.

Numerous attempts have been made to beneficially affect the course of pellagra by transfusion.

These attempts have been made without any well-defined idea as to how benefit would occur, if at all. Cole has published the most satisfactory reports. The procedure does not seem to be a rational one outside its general tonic effect.

### Miscellaneous Affections.

There appears to be no evidence that transfusion influences favorably leukemia, pernicious anemia, carcinoma, toxemia, certain drug poisons, acute hyperthyroidism or uremia.

In sarcoma there is some evidence of good resulting from transfusion though this has not yet been satisfactorily proven.

The results of transfusion for tuberculosis have been encouraging.

Cases of chronic suppuration are benefitted and rendered safe surgical risks.

In selecting a suitable donor a person in good health and a near relative, if possible, should be chosen. Where there is time—it requires 20 hours—hemolytic tests should be made. Dissimilar blood, that is, blood of another species, is dangerous. In some individuals, when similar blood is transfused, hemolysis is rapid and a violent reaction results. However, clinical experience has taught us that whatever hemolytic action ordinarily occurs does not impair the good results obtained.

The agglutinins may be disregarded.

